# Common occurrence of Belerina virus, a novel paramyxovirus found in Belgian hedgehogs

**DOI:** 10.1038/s41598-020-76419-1

**Published:** 2020-11-09

**Authors:** Bert Vanmechelen, Valentijn Vergote, Michelle Merino, Erik Verbeken, Piet Maes

**Affiliations:** 1 Laboratory of Clinical and Epidemiological Virology, Department of Microbiology, Immunology and Transplantation, Rega Institute for Medical Research, KU Leuven-University of Leuven, Herestraat 49, Box 1040, 3000 Leuven, Belgium; 2 Laboratory of Translational Cell and Tissue Research, Department of Imaging and Pathology, KU Leuven-University of Leuven, Herestraat 49, Box 7003 24, 3000 Leuven, Belgium

**Keywords:** Taxonomy, Viral epidemiology, Viral evolution, Viral reservoirs

## Abstract

Common or European hedgehogs can be found throughout Western Europe. They are known carriers of a variety of parasitic and bacterial pathogens, and have also been shown to carry several viruses, including morbilli-like paramyxoviruses, although the pathogenic and zoonotic potential of some of these viruses has yet to be determined. We report here the discovery of a novel paramyxovirus in Belgian hedgehogs, named Belerina virus. The virus was detected by nanopore sequencing of RNA isolated from hedgehog tissue. Out of 147 animals screened in this study, 57 tested positive for Belerina virus (39%), indicating a high prevalence of this virus in the Belgian hedgehog population. Based on its divergence from other known paramyxovirus species, Belerina virus is thought to represent a new species in the family *Paramyxoviridae*. Phylogenetic analysis groups Belerina virus together with the bat-borne Shaan virus within the genus *Jeilongvirus*, although expanding the tree with partial genomes shows Belerina virus forming a separate subclade within this genus, alongside a yet-unnamed paramyxovirus isolated from a greater tube-nosed bat. In summary, we discuss the complete genome sequence of Belerina virus, a putative new paramyxovirus species commonly found in Belgian hedgehogs.

## Introduction

European hedgehogs (*Erinaceus europaeus*), also known as common hedgehogs, can be found throughout Western Europe, stretching from southern Italy to southern Scandinavia and including the Iberian Peninsula^1^. They can live in a variety of habitats and are often found in gardens and parks. In recent years, hedgehogs have gained popularity as household pets, especially in the Western world, although this typically concerns four-toed hedgehogs (*Atelerix albiventris*), as the keeping of European hedgehogs is illegal in most Western countries^2–4^. Furthermore, wild hedgehogs that are underweight or have been injured are often housed by people aiming to help them regain their strengths and are sometimes kept as pets once they have recovered.

Because of this close contact between humans and hedgehogs, some concerns have arisen in recent years about the potential of zoonotic diseases to be transferred from hedgehogs to humans^4^. European hedgehogs are known to carry a variety of bacterial (Salmonella, *Yersinia Pseudotuberculosis* …) and fungal (Trichophyton, Microsporum) pathogens, and several cases of hedgehog-transferred zoonoses have been reported in the past decade^5–9^. Additionally, hedgehogs also harbor several zoonotic viruses, including rabies and tick-borne encephalitis virus, as well as other viruses of which the pathological and zoonotic potential remains to be fully elucidated^10–13^. An example of this is the finding of a paramyxovirus in European hedgehogs in 1981^14^.

The family *Paramyxoviridae* is a family of single stranded, negative-sense RNA viruses that was recently divided into four subfamilies^15^. The largest of these subfamilies, the *Orthoparamyxovirinae*, is further subdivided into eight genera and comprises 34 recognized species^16^. In addition to fish and reptiles, members of this subfamily infect a wide variety of mammals^17^. Most orthoparamyxoviruses are known to cause disease in their respective hosts and some (Hendra virus, Nipah virus) are known zoonotic pathogens that can spread to humans^18,19^. Based on a neutralization assay using antisera against different paramyxoviruses, the aforementioned virus discovered by Vizoso and Thomas was thought to belong to the Morbillivirus group (now genus *Morbillivirus*, subfamily *Orthoparamyxovirinae*)^14^. The virus was isolated from the feces and lungs of a severely ill hedgehog and caused a symptomatology similar to Canine distemper virus, although it was also found in feces of multiple healthy animals. Unfortunately, the genome sequence of this virus was not determined.

Here we report the complete genome sequence of Belerina virus, a putative new paramyxovirus species discovered in *Erinaceus europaeus*, which was detected in the context of a study aimed at virus discovery in different Belgian Eulipotyphla species. Belerina virus shows a relatively high prevalence in the hedgehogs screened in this study and appears to share similarities with paramyxoviruses found in bats.

## Results

### Discovery of a novel paramyxovirus species

We decided to use the Oxford Nanopore MinION to screen the total RNA, taken from the kidney of a European hedgehog, for the presence of previously undiscovered viruses. After running for 48 h, 5.1 M reads were produced, totaling 1.25 Gb after trimming. A tblastx search of all reads against the viral subset of the NCBI RefSeq database found 38 reads displaying limited similarity to different paramyxovirus genomes, indicating the presence of a novel paramyxovirus. Based on these 38 reads, we designed primer sets spanning the entirety of the viral genome and used these to determine the complete genome sequence of this virus, through a combination of PCR and 5′/3′ RACE, and subsequent Sanger sequencing. The complete genome (GenBank: MN561699) is 15,948 nucleotides long, thereby adhering to the so-called ‘rule-of-six’, which states that efficient replication of paramyxovirus genomes is dependent on their length being a multiple of six^20^. This new virus was given the name ‘Belerina virus’, based on its location of origin (Belgium) and the identity of its host (*Erinaceus Europaeus*).

### Phylogenetic analysis

**Figure 1:**
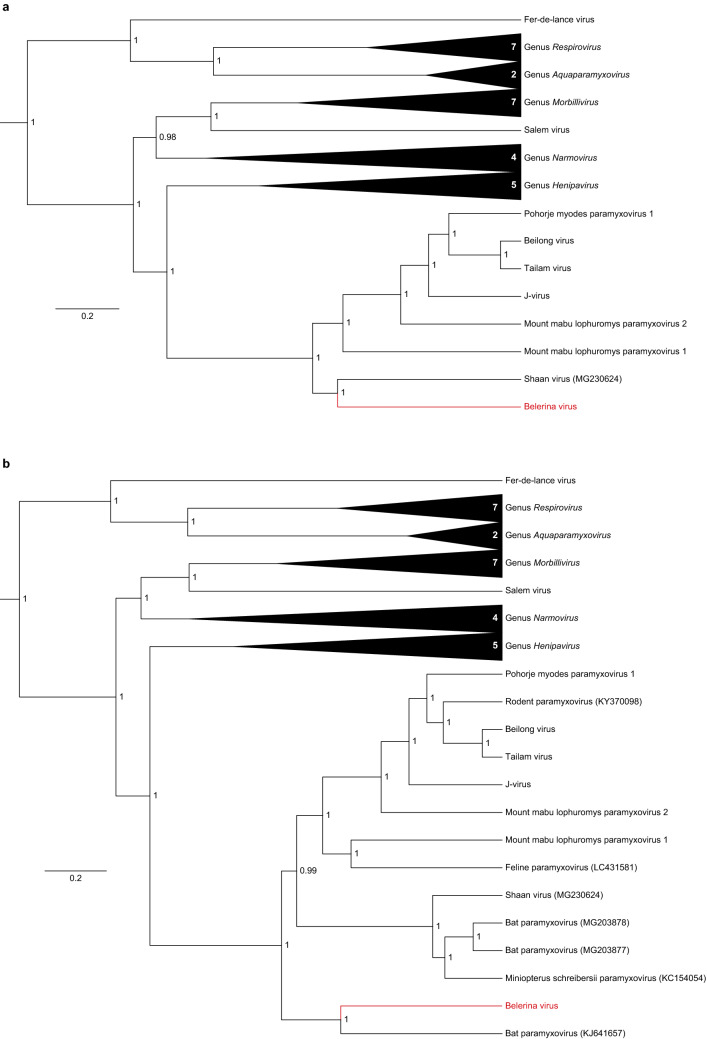
Maximum clade credibility tree of the subfamily *Orthoparamyxovirinae*. Phylogeny was imputed from concatenated alignments of the N, P, M, F, G and L proteins of all orthoparamyxoviruses. **(a)** A tree made using only Belerina virus and the 34 currently accepted species shows Belerina virus clustering alongside Shaan virus within the genus *Jeilongvirus*. **(b)** A more detailed tree incorporating Belerina virus and all 40 currently available (near-)complete orthoparamyxovirus genomes shows that together with the bat paramyxovirus BtMl-ParaV (KJ641657), Belerina virus forms a sister clade to all currently known jeilongviruses. For all recognized species, the RefSeq sequence was used to generate the tree. Accession numbers of unclassified/partial genomes are denoted at each branch. Values at the nodes indicate posterior support for each cluster. Branch lengths represent the number of amino acid substitutions per site. FigTree v1.4.3 (https://tree.bio.ed.ac.uk/software/figtree/) was used to draw the trees.

Bayesian-based phylogenetic analysis of all 34 currently recognized orthoparamyxovirus species, based on the amino acid sequence of the six major paramyxovirus ORFs (N, P/V/C, M, F, G and L), groups Belerina virus within the genus *Jeilongvirus* (Fig. 1a). This genus currently contains seven species, six rodent-borne paramyxoviruses and one paramyxovirus that was found in bat urine. Together with this bat-borne Shaan virus, Belerina virus seems to form a sister clade to the rodent-borne jeilongviruses. Interestingly, if the tree is expanded with all available complete and near-complete (> 90%) orthoparamyxovirus genomes, including several viruses isolated from different bat species, it becomes apparent that Belerina virus is phylogenetically most closely related to the bat paramyxovirus BtMl-ParaV (KJ641657). Together with this bat virus, Belerina virus forms a sister clade to all other jeilongviruses, including the other bat viruses (Fig. 1b). As a consequence of the incorporation of partial genomes into the analysis, the alignment on which the tree is based needed to be trimmed at both ends, resulting in 3845 informative sites, compared to 4344 for the tree shown in Fig. 1a. However, as illustrated in Supplementary Fig. S1, the altered topology of the tree in Fig. 1b is independent of this alignment trimming and is caused by the addition of the partial genomes, highlighting the importance of their inclusion in the phylogenetic analysis.

### Genome organization of Belerina virus

Members of the family *Paramyxoviridae* typically share a similar genome organization, characterized by six major ORFs. These ORFs encode the nucleocapsid (N), a phosphoprotein (P), a matrix protein (M), two membrane glycoproteins (F and G) and the viral polymerase (L). Through a combination of leaky scanning (C) and mRNA editing (V, W), some additional accessory proteins are usually expressed from the P ORF. The V, W RNA editing site is marked by a motif sequence (YTAAAARRGGCA) that is conserved across all members of the genera *Henipavirus*, *Morbillivirus*, *Jeilongvirus* and *Narmovirus*, with the exception of Cedar virus and feline morbillivirus. Also in the genome of Belerina virus, this motif sequence is present (TTAAAAAAGGCA), indicating that mRNA editing likely also occurs in the case of Belerina virus, although this was not verified experimentally.

A defining characteristic of members of the genus *Jeilongvirus* is that their genomes contain additional ORFs between the F and G ORFs. In the genomes of Mount Mabu lophuromys paramyxovirus 1/2 (MMLPV-1/-2), as well as in those of the yet-to-be-classified feline paramyxovirus and the unnamed bat paramyxovirus BtMl-ParaV (KJ641657), only one such ORF is present, encoding a transmembrane protein (‘TM’). In all other jeilongviruses, a second additional ORF is present, encoding a small hydrophobic protein (‘SH’). The role of these two proteins is not yet fully elucidated, although recent research has shown that for JV, the SH protein is involved in inhibiting the production of TNF-α, while the TM protein seems to stimulate cell-to-cell fusion^21–23^. A SH protein is also present in a limited number of other paramyxoviruses (Avian avulavirus 6, Mammalian rubulavirus 5, Mojiang henipavirus, Mumps rubulavirus and Bat mumps rubulavirus), while the TM protein is found exclusively in members of the genus *Jeilongvirus*. The genome of Belerina virus encodes only the TM protein, although this protein is slightly larger (310 aa) than the TM proteins (218–275 aa) of other jeilongviruses.

### Occurrence and spread in Belgium

**Figure 2:**
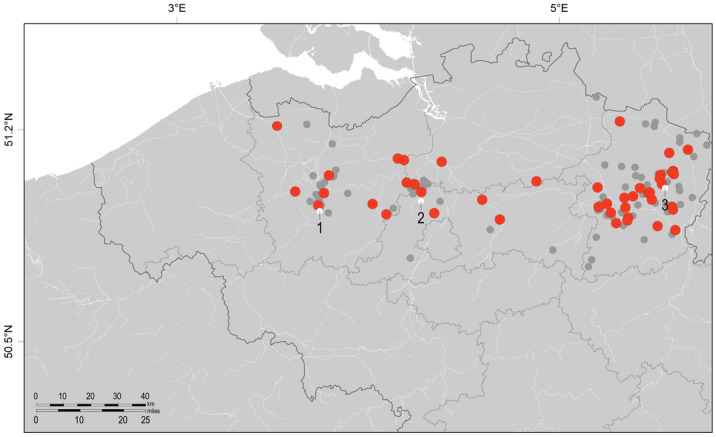
Map of Belgium detailing the towns of origin of all collected hedgehogs. Each circle denotes one animal. Animals testing positive for the presence of Belerina virus RNA are marked in red. The white dots indicate the animal shelters that collected the animals. 1 = Merelbeke, 2 = Malderen, 3 = Opglabbeek. Map drawn using Adobe Photoshop CC 2019 (https://www.adobe.com/be_nl/products/photoshop.html).

We collected kidney tissue samples from 147 Belgian hedgehogs that were brought in to three different animal rescue shelters. In total, 57 animals tested positive for Belerina virus (38.78%). A map showing the origin of the different animals is shown in Fig. 2. Most samples originated from the east side of Flanders, where the largest Belgian animal rescue shelter is located (Natuurhulpcentrum vzw, Opglabbeek). Belerina virus was detected in animals from all three collection points, although a formal comparison of positivity rates is hindered by the disequilibrium in the amount of animals collected at the different sites.

### Histological analysis

The animals collected during the last year of sample collection (10) were selected for later histological analysis. A detailed overview of the histological findings is shown in Supplementary Table S2. Two of these animals (animals 7 and 10) tested positive for Belerina virus and six animals were infected with lungworms (animals 2–7). The lungs of all animals had alveolar edema. The interstitial tissue of animal 10 showed only agonal lesions, while that of animal 7 displayed signs of pneumonia in the form of follicular hyperplasia and the presence of granulomas. However, this pneumonia is unlikely to be of viral origin, as two of the virus-negative animals (animals 2 and 4) displayed similar lesions. With the exception of the virus-negative animals 1 and 5 showing acute tubular necrosis, no kidney lesions were observed in any of the animals. Taken together, we observed no Belerina-virus specific pathology in the kidneys or lungs of the observed animals.

## Discussion

**Figure 3:**
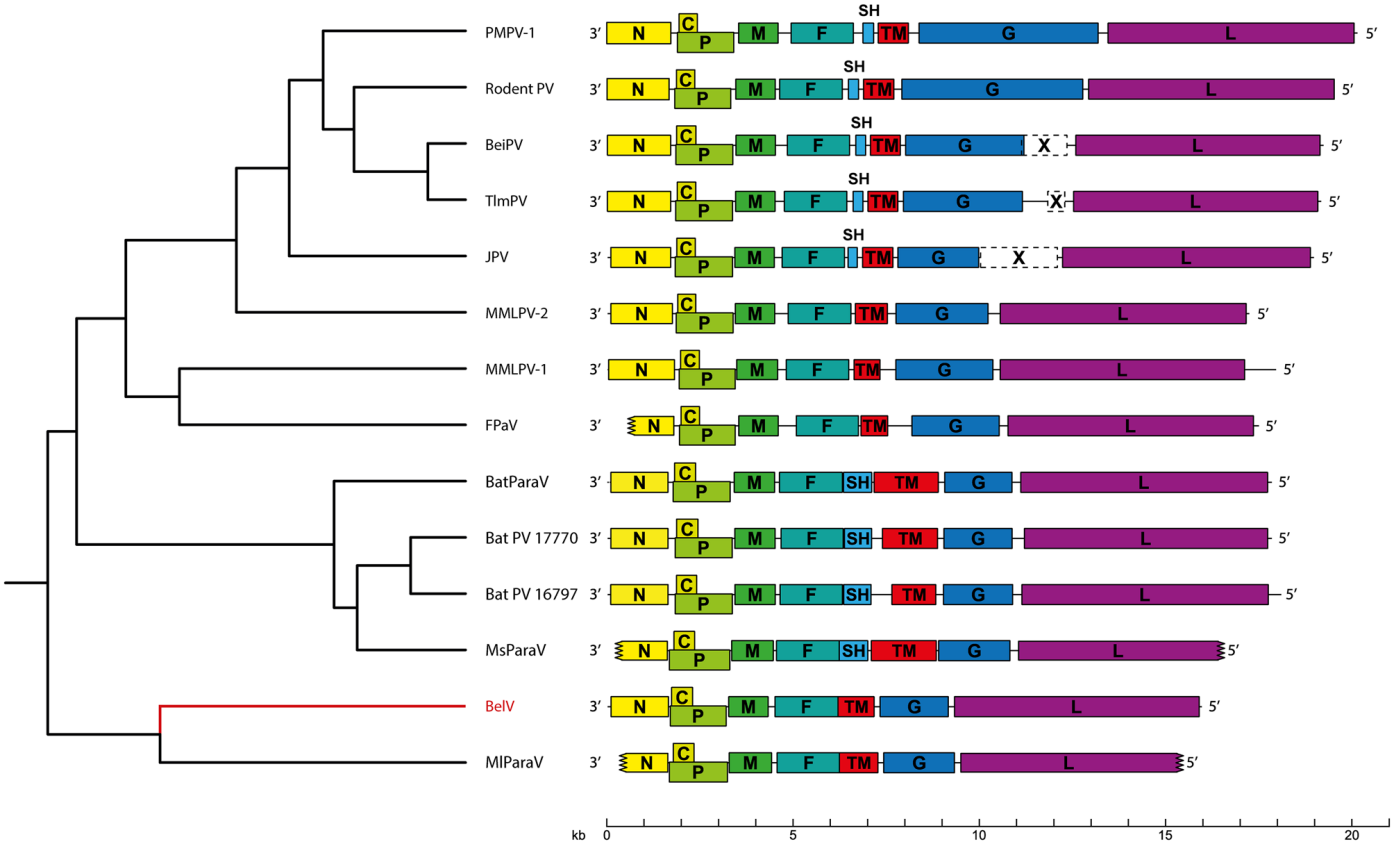
Genome organization of all (putative) jeilongviruses. The TM gene is a characteristic feature of jeilongviruses and is not found in other paramyxoviruses. The SH gene is also predominantly found in members of the genus *Jeilongvirus*, but is absent in some members, including Belerina virus. The tree on the left side is taken from Fig. 1b and denotes the phylogenetic relationship between the different viruses. Figure drawn using Adobe Illustrator CS6 (https://www.adobe.com/be_nl/products/illustrator.html).

In this study, we present the complete genome sequence of Belerina virus, a putative new paramyxovirus species found in European hedgehogs. Belerina virus clusters together with the bat-borne Shaan virus, forming a sister clade to the other members of the genus *Jeilongvirus*. Interestingly, Belerina virus does not encode an SH protein, like Shaan virus, but instead has the same unique genome structure as MMLPV-1/-2, containing seven major ORFs (N-P/V/C-M-F-TM-G-L). The phylogenetically basal position of these viruses, relative to other members of the genus *Jeilongvirus*, seems to suggest that jeilongviruses have acquired their additional ORFs sequentially, starting with the acquisition of the TM ORF, followed later by the addition of the SH ORF. Additionally, this SH-ORF acquisition appears to have occurred twice, once in the rodent-borne jeilongvirus lineage and once in the case of Shaan virus. When the phylogenetic analysis is expanded to also include likely paramyxovirus species for which only partial, yet largely complete (> 90%), genomes are known: rodent paramyxovirus, feline paramyxovirus and four additional (partial) genomes of bat paramyxoviruses (Bat PV 16797, Bat PV 17770, BtMf-ParaV and BtMl-ParaV), Belerina virus now clusters with BtMl-ParaV, but separately from the other bat jeilongviruses. This clustering is also reflected in the genome organization of these viruses, with both Belerina virus and BtMl-ParaV lacking an SH ORF, while the four other bat virus genomes do presumably encode SH proteins, although both these SH proteins and these viruses’ TM proteins are significantly larger than the SH and TM proteins of other jeilongviruses (Fig. 3). Important to note is that the SH proteins of these four bat viruses and the SH proteins of other jeilongviruses share no significant sequence similarity. This seems to suggest that the acquisition of the SH ORF has occurred separately in two different lineages of jeilongviruses, assuming, as is the case for SH proteins of other paramyxoviruses, that the SH proteins of these different viruses are functional homologs.

Another interesting feature of jeilongviruses that sets them apart from other paramyxoviruses is the exceptional size of their attachment glycoprotein (‘G’). The paramyxovirus attachment glycoprotein, sometimes designated H(N) protein if the glycoprotein possesses hemagglutination (and neuraminidase) activity, is primarily involved in regulating the binding of virus particles to target cells^17^. In all non-jeilongvirus paramyxoviruses, it has a length of ~ 600 amino acids, the majority of which form a large ectodomain that is present on the outside of the viral membrane. For jeilongviruses, however, the size of the G protein is highly variable and can reach lengths of up to ~ 1600 amino acids. Moreover, although these expansions of the G protein share no significant sequence similarity between different jeilongviruses, they do have a certain degree of structural homology, as they are all characterized by a high fraction of serine, threonine and proline^24^. The G protein of Belerina virus, however, is only 597 amino acids long, and as such lacks this S/T/P-rich expansion of the ectodomain. Interestingly, the five bat jeilongviruses also lack this expansion of the G protein ectodomain, further illustrating the separate evolutionary history of the rodent-borne jeilongviruses. Based on these observations, it is clear that, even though members of the genus *Jeilongvirus* have several unique characteristics that set them apart from other paramyxoviruses, only the presence of a TM ORF is a characteristic shared by all jeilongviruses, while the occurrence of an SH ORF or the expansion of the G ORF seem to be limited to certain subclades. Based on the uniqueness of these subclades, it has previously been suggested that the genus *Jeilongvirus* could be split into separate genera, with Shaan virus representing a separate genus, tentatively called ‘Shaanvirus’^25,26^. However, even though there are clear differences between the different subclades, the genetic distance between all jeilongviruses is somewhat limited and seemingly insufficient to warrant the establishment of separate genera, especially when compared to the within-genus genetic distance of other genera.

For this study, 147 animals from different locations throughout Belgium were screened for the presence of Belerina virus. Almost 40% of these animals tested positive. It is possible that this number is an overestimation of the actual prevalence due to sampling bias. The animals tested in this study were all brought into animal rescue shelters because they were wounded, weakened, sick or dead. If Belerina virus actually causes disease in hedgehogs, this method of sample collection could result in an overestimation of virus prevalence in the Belgian hedgehog population. However, histological analysis of two virus-positive animals revealed no virus-specific kidney or lung lesions, hinting at a limited or sporadic pathogenicity (if any) of Belerina virus. Unfortunately, we were unable to acquire additional tissue samples for more extensive histological analyses. Until now, there has been only one report of paramyxoviruses causing disease in hedgehogs. In their report, Vizoso and Thomas describe the finding of a paramyxovirus in a hedgehog displaying symptoms akin to canine distemper^14^. Based on neutralization assays, they concluded that the virus they found was most similar to viruses of the Morbillivirus group (now genus *Morbillivirus*, subfamily *Orthoparamyxovirinae*). It is also stated briefly that similar viruses could be isolated from apparently healthy hedgehogs. Based on the information given in their report, it is possible that the virus they described belongs to the same species as Belerina virus, but this could not be verified due to a lack of sequence information.

In summary, we report here the first description of Belerina virus, a novel paramyxovirus found in European hedgehogs. Belerina virus appears to have limited pathogenicity and seems to be prevalent in the Belgian hedgehog population. Comparison of the genome organization as well as phylogenetic analyses indicate that Belerina virus is most similar to a bat virus for which only a partial genome sequence is available, but that together these two viruses represent a separate clade within the genus *Jeilongvirus*.

## Materials and methods

### Sample collection

A total of 147 hedgehog carcasses were collected from 2015–2019 from three animal shelters in Belgium (Fig. 2). All hedgehogs were brought in from surrounding towns because they were wounded, weakened, sick or dead. Animals that could not be helped were euthanized at the animal shelter. Carcasses were stored at − 20 °C prior to dissection. Heart, lungs, kidneys and spleen were removed aseptically from each animal and stored in RNAlater Stabilization Solution (Ambion, Thermo Fisher Scientific, Waltham, MA, USA) overnight, after which the RNAlater was removed and the organ stored at − 80 °C. Ten animals were dissected on site and their lungs and kidneys harvested immediately, with one set of organs being stored in RNAlater for RNA extraction and the other in formaldehyde (37%) for later histological analysis. Approval for the collection and handling of material of a protected animal species was given by the “Agentschap voor Natuur en Bos-Cel Gebiedsgericht- en soortenbeleid”, case number ANB/BL/FF-V13-00172.

### Discovery of Belerina virus by nanopore sequencing

Viral RNA was extracted from the kidney of one hedgehog by homogenizing 20 mg of renal tissue in 100 µl sterile PBS, using zirconium oxide beads, for two minutes at 4000 rpm using a Minilys homogenizer (Bertin Technologies, Montigny-le-Bretonneux, France). The resulting homogenate was centrifuged at 17.000 *g* for 3 min and the supernatant passed through a 0.8 μm centrifugal filter with a hydrophilic polyvinylidene fluoride membrane (EMD Millipore, Burlington, MA, USA). Further viral enrichment was achieved by layering 10 ml of filtered supernatant onto 2 ml of 30% (wt/vol) sucrose-PBS and centrifuging at 30,000 rpm for 3 h in an SW41 rotor (see Stang et al.)^27^. Following ultracentrifugation, total RNA was extracted using the QIAamp Viral RNA Mini kit (Qiagen, Leiden, The Netherlands). No carrier RNA was used. Any DNA present in the eluate was then removed enzymatically using TURBO DNase (2U; Thermo Fisher Scientific, Waltham, MA, USA). Following a clean-up step using the RNeasy MinElute Cleanup kit (Qiagen), according to the manufacturer’s instructions, the purified RNA was amplified using the Complete Whole Transcriptome Amplification Kit WTA2 (Sigma-Aldrich, St. Louis, MO, USA). The resulting cDNA was cleaned up using the MSB Spin PCRapace kit (Stratec, Birkenfeld, Germany) and used to prepare a MinION sequencing library using the SQK-LSK108 kit (Oxford Nanopore Technologies, Oxford, UK), following the manufacturer’s recommendations. The sequencing library was ran for 48 h on a FLO-MIN106 flowcell (Oxford Nanopore Technologies). Adapter sequences were removed using Porechop (v0.2.4)^28^.

### Finishing the Belerina virus genome by Sanger sequencing

Primers were designed, based on the sequences of the reads obtained by nanopore sequencing, to generate amplicons spanning the remaining gaps in the Belerina virus genome. Amplicons were generated using the OneStep RT-PCR kit (Qiagen). Used PCR conditions were: reverse transcription at 50 °C for 30 min, followed by 15 min at 95 °C and 40 cycles of 30 s at 94 °C, 30 s at 55 °C and 1 min at 72 °C, and a final extension step at 72 °C for 10 min. The resulting amplicons were purified using PureIT ExoZAP (Ampliqon, Odense, Denmark) and sent to Macrogen (Macrogen Europe, Amsterdam, The Netherlands) for Sanger sequencing. Given the low single read accuracy of the nanopore data, the obtained Sanger sequences were used to design new primer sets, spanning the gaps between the previously obtained amplicon sequences, allowing for the resequencing of all used nanopore data. PCR cycling and Sanger sequencing was done as described above. All used primers are listed in Supplementary Table S1. All chromatogram files were inspected using Chromas (v2.6.2) and joined using Seqman (v7.0.0).

### RACE

Determining the exact 5′ and 3′ ends of the Belerina virus genome was done using the 5′/3′ RACE kit 2nd generation (Roche, Mannheim, Germany), replacing the standard Oligo dT-Anchor Primer by a slightly modified one that also targets the conserved trinucleotide found at the 3′/5′ ends of paramyxovirus genomes (GAC CAC GCG TAT CGA TGT CGA CTT TTT TTT TTT TTT ACC). For the 3′ end, a poly-A tail was added to the virus genome prior to cDNA generation, using the Poly(A) Polymerase Tailing Kit (Epicentre, Madison, WI, USA) according to the manufacturer’s instructions. For both ends, amplification of the poly-A tailed RNA/cDNA was done with the OneStep RT-PCR kit (Qiagen). See Supplementary Table S1 for a list of the used primers. PCR cycling and Sanger sequencing was performed as described above.

### RNA extraction and PCR screening of Belerina virus

For all 147 hedgehogs, viral RNA was extracted from one of the kidneys using the RNeasy Mini kit (Qiagen), according to the manufacturer’s protocol. All extracts were screened for the presence of Belerina virus using a specific primer set targeting a 214 bp region in the Belerina virus L gene (Supplementary Table S1). PCR amplification was done with the OneStep RT-PCR kit (Qiagen), using the following cycling conditions: 30 min at 50 °C, 15 min at 95 °C, 40 cycles of 30 s at 94 °C, 30 s at 54 °C and 1 min at 72 °C, and a final extension step of 10 min at 72 °C.

### Phylogenetic analysis

The complete amino acid sequences of the N, P, M, F, G and L open reading frames (ORF) of all publicly available complete and near-complete orthoparamyxovirus genomes were aligned separately using MAFFT (v7.123b) and subsequently concatenated^29^. The resulting alignment was trimmed using trimAL (v1.4.rev15) with the gappyout setting^30^. Following selection of an LG + G + I model, using ProtTest (v3.4.2), as an adequate model to describe the amino acid substitution process, BEAST v1.10.4 was used to infer Bayesian phylogenetic trees^31–33^. Markov chain Monte Carlo analyses were ran until adequate effective sample sizes (ESS > 200) were obtained. TreeAnnotator was used to summarize a maximum clade credibility tree from the posterior tree distribution, employing a burn-in of 10%. FigTree v1.4.3 was used to visualize the resulting tree.

## Supplementary information

Supplementary Information.

## Data Availability

The complete Belerina virus genome sequence has been submitted to GenBank (accession number: MN561699).
