# Supplementary material for: Common occurrence of Belerina virus, a novel paramyxovirus found in Belgian hedgehogs
Source: Sci Rep. 2020 Nov 9;10:19341. doi: 10.1038/s41598-020-76419-1 (PMC7653956; doi:10.1038/s41598-020-76419-1)
Supplement: Supplementary file 1 — Supplementary Information. [file 41598_2020_76419_MOESM1_ESM.pdf]

Supplementary Information

Common occurrence of *Belerina virus*, a novel paramyxovirus found in Belgian hedgehogs

Bert Vanmechelen, Valentijn Vergote, Michelle Merino, Erik Verbeken and Piet Maes

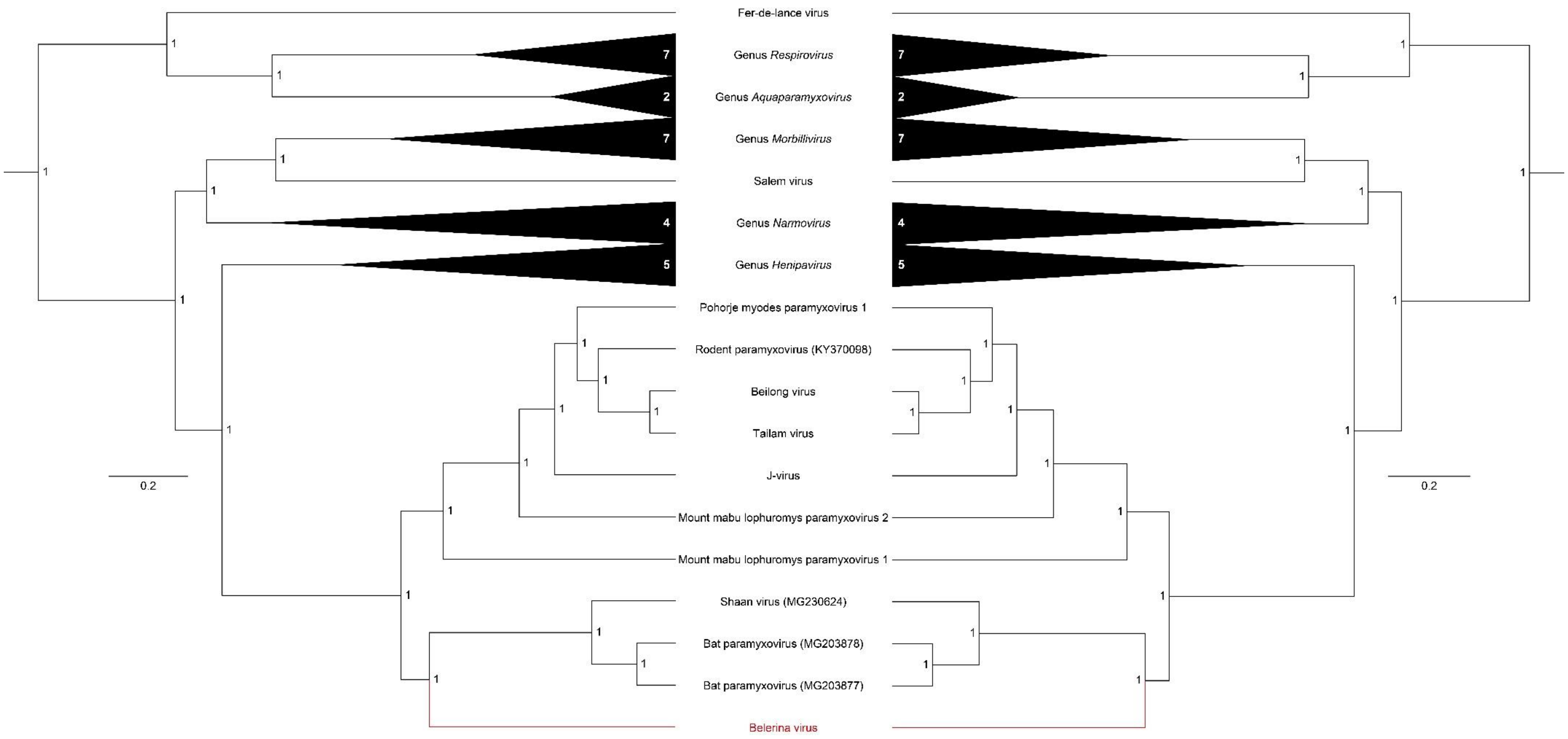

Supplementary Figure S1. Alignment trimming does not impact the tree topology. Phylogenetic trees made based on alignments of the deduced amino acid sequences of the six major paramyxovirus ORFs (N-P-M-F-G-L) of all known coding-complete orthoparamyxovirus genomes show the exact same topology when using the full alignment (left) or when trimming ~500 sites from the alignment (right), reflecting the tree shown in Figure 1b, indicating that the altered tree topology in Figure 1b can be attributed to the inclusion of partial genomes and not to differences in the alignment trimming.

Supplementary Table S1: Primers used for PCR and Sanger sequencing.

Name	Sequence
1F	GAGATTCTGAAAGTGAAGGCATAA
1R	GCGAATTTTGCTTGTGCAG
2F	TGGCAGGGTTCTTCCTAACA
2R	TCTTGAAGGATGGTGTTTGCT
3F	GGTCCCAGCACCAACCTAC
3R	TCAGCTAGACCTCGCCTGTT
4F	TCCTAGGAAATCAAGCCAAAA
4R	TCAACTCCACGCTACTGCAT
5F	CCCCCGAGAGTCTTCTTGAG
5R	TGATCAAGGTATGGTGAGAGCA
6F	AAGCAAGCTTTGGTGAGCAT
6R	TACAGGCATCCACTCCACAC
7F	GCCTTTAATGGCGATTTTGA
7R	CATTACGACCCATGTTGACG
8F	AGCAGCTTAATTCCCCAAGA
8R	GGGTGGGACTGGTGTTGT
9F	TGCTGATTACCTGGCTGCTA
9R	TGCGATTAAGACCATCATTCA
10F	TTTTCACTGGGAGCACAGG
10R	CGGGGTCATCTCGACAATAG
11F	TGATGCCTTTCCTCTTATCTCC
11R	CACTTCGACGCCTGCATAAC
12F	CAACATGCGAGATGATGATG
12R	AAACCCATAAAACAGCCAAA
13F	AAGGCCCATGCATTATTCTG
13R	AATCTTGACTCCCGCTTCAT
14F	GGACCCTCACTGTCCACCTA
14R	CCCTTGGGATAATGATAGGC
15F	CGATTGGGCTAGTGATCCAT
15R	CATTTCCTTAAGGCAGCAGTG
16F	TAATATTGCCCACCGCCTTA
16R	GGTCTGTGAGAACGGTGCAT
17F	TTGCGGCGCTTATCAGTTAT
17R	GCTGTGGAGTGTAATCATTGG
18F	CACATGGATTGGTTCACTCG
18R	CCCAGAAGTTGTGGTCTTGG
RACE Anchor primer	GACCACGCGTATCGATGTCGACTTTTTTTTTTTTTTTTACC
SP1	GTACACAGTGAGGTTAGGAGG
SP2	GATTTAAAGGATAATTCCTGC
SP3	GGGAGAAATATGCTAGGACTGGG
SP4	CCCCTTTAACAGGTGCATTCTTG
SP5	GCGTTGGATCTGTTGATG
Screening primer F	TCGCAGAGTCACTAATAGCC
Screening primer R	TGCTCTGAGTTACTTGAATGG

Supplementary Table S2: Overview of observed histological lesions.

				Kidney	Lung						
Animal	Prior injury/sickness	Euthanized	Belerina virus	Acute tubular necrosis	Worms		Bronchitis	Alveolar edema	Alveolar pneumonia	Interstitial pneumonia	Signs of shock
					Alveolar	Bronchial					
1	Unknown	-	-	+	ND	ND	ND	ND	ND	ND	ND
2	Injured	+	-	-	+	+	-	+	+	+	-
3	Injured	+	-	-	-	+	-	+++	-	-	-
4	Sickness	+	-	-	-	+	+	+	+	+	-
5	Sickness	-	-	+	-	+	+	+	-	-	+
6	Sickness	-	-	-	-	+	-	++	-	-	+
7	Injured	+	+	-	-	+	-	+	+	+	-
8	Sickness	-	-	-	-	-	-	++	-	-	+
9	Injured	-	-	-	-	-	-	+++	-	+	+
10	Injured	-	+	-	-	-	-	+	-	-	+

ND = not determined
